# QualComp: a new lossy compressor for quality scores based on rate distortion theory

**DOI:** 10.1186/1471-2105-14-187

**Published:** 2013-06-08

**Authors:** Idoia Ochoa, Himanshu Asnani, Dinesh Bharadia, Mainak Chowdhury, Tsachy Weissman, Golan Yona

**Affiliations:** 1 Department of Electrical Engineering, Stanford University, Stanford, CA, USA

**Keywords:** Next generation sequencing, Quality scores, Compression, FASTQ format, Rate distortion, Mean squared error

## Abstract

**Background:**

Next Generation Sequencing technologies have revolutionized many fields in biology by reducing the time and cost required for sequencing. As a result, large amounts of sequencing data are being generated. A typical sequencing data file may occupy tens or even hundreds of gigabytes of disk space, prohibitively large for many users. This data consists of both the nucleotide sequences and per-base quality scores that indicate the level of confidence in the readout of these sequences. Quality scores account for about half of the required disk space in the commonly used FASTQ format (before compression), and therefore the compression of the quality scores can significantly reduce storage requirements and speed up analysis and transmission of sequencing data.

**Results:**

In this paper, we present a new scheme for the lossy compression of the quality scores, to address the problem of storage. Our framework allows the user to specify the rate (bits per quality score) prior to compression, independent of the data to be compressed. Our algorithm can work at any rate, unlike other lossy compression algorithms. We envisage our algorithm as being part of a more general compression scheme that works with the entire FASTQ file. Numerical experiments show that we can achieve a better mean squared error (MSE) for small rates (bits per quality score) than other lossy compression schemes. For the organism *PhiX*, whose assembled genome is known and assumed to be correct, we show that it is possible to achieve a significant reduction in size with little compromise in performance on downstream applications (e.g., alignment).

**Conclusions:**

QualComp is an open source software package, written in C and freely available for download at *https://sourceforge.net/projects/qualcomp*.

## Background

It has been more than a decade now since the first draft of the human genome was published [1]. The Human Genome Project, which required a significant collaborative effort of many scientists for more than 10 years, was completed using the Sanger sequencing technology. Just a decade later, many medium and small size laboratories achieve the task of sequencing complete mammalian genomes within a few weeks using the new Next Generation Sequencing (NGS) technologies. The cost per genome has decreased from $100M in 2001 to $10K in 2012 [2]. Current sequencers are capable of generating close to tera-base worth of data that needs to be stored and processed. Several recent studies, such as the cow rumen [3] and the MetaHit [4] metagenomic projects resulted in hundreds and hundreds of gigabase worth of datasets. As project scales continue to grow, it is expected that the bottleneck will move towards the computational aspects, in particular with respect to the analysis and storage of the data. For example, there is an explosive growth of data submitted to the freely available next-generation sequence data archive, the Sequence Read Archive (SRA) [5]. Thus compressing this data will facilitate its storage and dissemination.

Several methods are used by the different NGS technologies for the readout of the sequencing signal (known as base calling). This process may be affected by various factors, which may lead to a wrong readout of the sequencing signal. In order to assess the probability of base calling mistakes, the sequencers generate, in addition to the nucleotide sequences (reads), quality scores that reflect the level of confidence in the readout of each base. That is, the higher the quality score, the higher the reliability of the corresponding base, and vice versa. Specifically, the quality score *Q* is the integer mapping of *P* (the probability that the corresponding base call is incorrect) and can be represented in (at least) the following scales/standards: 

•*Sanger or Phred* scale [6]: $Q=-10\log_{10}P$.

•*Solexa* scale: $Q=-10\log_{10}\frac{P}{1-P}$.

Different NGS technologies use different scales, *Phred + 33*, *Phred + 64* and *Solexa + 64* being the most common ones. For example, *Phred + 33* corresponds to values of *Q* in the range [33:73].

Quality scores are important and very useful in many downstream applications such as trimming (used to remove untrusted regions) [7,8], alignment [9-12] or Single Nucleotide Polymorphism (SNP) detection [13,14], among others. However, they significantly increase the size of the files storing raw sequencing data.

The literature abounds in efforts to compress genomic data. Whole genome level compression without the aid of any external information has been the focus of [15-19] and references therein. More recent contributions show that further compression can be achieved by mapping the target genome to a reference genome, and encoding only the differences between the two [20-27]. Compression of raw sequencing data has also been studied in the literature. While the compression scheme proposed in [28] focuses only on the nucleotide sequences, most approaches consider the compression of the entire file, including the quality scores [29-37].

In this paper, we concentrate on the lossy compression of the quality scores, as they take up a significant chunk of the storage space. FASTQ files are widely accepted as a standard for storing sequencing data, and are the ones considered in this paper. FASTQ files consist of separate entries for each read, each consisting of four lines. The first one is for the *header line*, which begins with the ‘@’ character and is followed by a sequence identifier and an optional description. The second one contains the *nucleotide sequence*. The third one starts with the ‘+’ character and can be followed by the same sequence identifier and optional description as the first line. Finally, the fourth line is for the *quality scores* (encoded in ASCII) associated with the bases that appear in the nucleotide sequence of line two (both lines must contain the same number of symbols). The following is an example of a FASTQ file corresponding to a read of length 26 and quality scores in the scale *Phred + 33*.

@SRR001666.1GATTTGGGGTTCAAAGCAGTGCAAGC+IIIHIIHABBBAA=2))!!!(!!!((

Unlike header lines and nucleotide sequences, quality scores are particularly difficult to compress, due to their higher entropy and larger alphabet. The importance of lossy compression in the context of reducing storage of sequenced data has been highlighted in [38]. Although discarding information may not always be welcome, several lossy compression schemes for the quality scores have been proposed recently in the literature as a partial solution to reduce the storage space of genomic data. It has been presented as a plausible idea in [5] and as a concrete algorithm as part of the SLIMGENE package in [32]. The SLIMGENE package considers the compression of both the nucleotide and quality score sequences, and it includes a module which performs lossy compression of quality scores based on fitting a fixed state markov model on adjacent quality scores as well as on reducing the alphabet. They use SNP variant calling as their performance metric and show that lossy compression has a minimal effect on performance. In [33], a metric called “quality-budget” is used to selectively discard the quality scores which match perfectly to the reference, with only quality scores with sufficient variation being retained. Cramtools [36] is a software built on this principle of reference-based compression that also allows lossy compression of the quality scores. Recently, [39] considered relative mapping accuracy of the reads as the metric and applied various lossy transform techniques to show that impressive compression can be achieved with a small loss in performance. The software fastqz [34] has the option of quantizing the quality scores for lossy compression. Finally, the SCALCE software [35] provides an optional controlled lossy transformation approach that reduces the alphabet size based on the observation that “neighboring” quality scores are similar in general.

Reducing the storage space of quality scores by performing lossy compression may affect the performance of downstream applications, and therefore minimizing the loss in performance is an important goal. However, due to the noisy nature of the several models/technologies that generate the quality scores and the varied use that different downstream applications make of them, it is difficult to design a compression scheme that achieves this goal independently of the technology and downstream application. Furthermore, it is not clear how to compare the different lossy compression schemes proposed so far in the literature, in the absence of a defined metric. In this work, we propose a scheme based on rate distortion theory that compresses the quality scores by allocating as many bits per quality score sequence as specified by the user, while minimizing a given distortion between the uncompressed (i.e., the original quality scores) and the reconstructed quality scores after the lossy compression. We choose a mathematical quantity for the distortion rather than a “physical distortion” or performance loss with respect to downstream applications, thus making the proposed scheme independent of the sequencing technology and the downstream analyzers.

Specifically, we use mean squared error (MSE) as the measure of performance for our lossy compression. We believe that reduced MSE translates to minimizing the incurred loss in the downstream applications. Our algorithm assumes that the quality scores are generated by a multivariate Gaussian. This is justified by the fact that, given a vector source with a particular covariance structure, the Gaussian multivariate source is the least compressible and, further, a code designed under the Gaussian assumption will perform at least as well on any other source of the same covariance [40]. Then, using the singular value decomposition (SVD) technique, we decorrelate the quality scores thereby getting a multivariate Gaussian characterized by a diagonal covariance matrix, or in other words, “independent” streams of quality scores. This allows us to use optimization techniques from rate distortion theory to optimally allocate bits to minimize the MSE. Because reads within a file may have very different qualities and the proposed method is based on the statistics of the quality score sequences, the proposed algorithm also allows the user to cluster the data prior to compression, to improve the statistics and hence the performance. We compare several lossy compression schemes based on this criterion, and see that our algorithm achieves much smaller MSE for small rates. Also, we show that our algorithm can work at rates not achieved by other lossy compression algorithms. We further show that, for a data set where the assembled genome is known and assumed to be correct, one can get performance in some downstream applications comparable to that achieved using the original (uncompressed) quality scores. Finally, our algorithm allows the user to specify the rate prior to compression, which as far as we know is the first implementation of lossy compression of quality scores that has this characteristic.

## Methods

### The compression method

We now formalize the problem of lossy compression of quality scores and describe the proposed method. As stated in previous sections, the FASTQ format is widely accepted as the standard to store sequencing data. Therefore, we consider the compression of quality scores presented in this format, and assume all the reads are of the same length *n* within a file. Denote the number of entries in the FASTQ file by *N*, where each entry is composed of four lines. The quality score sequences presented in a FASTQ file are denoted by ${\left\{Q_{i}\right\}}_{i=1}^{N}$, where **Q**_*i*_=[*Q*_*i*_(1),…,*Q*_*i*_(*n*)]. Our goal is to design an encoder-decoder pair that describes the quality score vectors using only as many bits as specified by the user, while minimizing a given distortion *D* between the original vectors ${\left\{Q_{i}\right\}}_{i=1}^{N}$ and the reconstructed vectors ${\left\{{\hat{Q}}_{i}\right\}}_{i=1}^{N}$. More specifically, we consider that each **Q**_*i*_ is compressed using at most *nR* bits, where *R* denotes the rate (bits per quality score), and that the distortion *D* is computed as the average distortion of each of the vectors, i.e., $D=\frac{1}{N}\sum_{i=1}^{N}D(i)$. Furthermore, we consider the MSE as our given distortion function $d:(Q,\hat{Q})\to R^{+}$, which operates symbol by symbol (as opposed to block by block), so that $D(i)=d(Q_{i},{\hat{Q}}_{i})=\frac{1}{n}\sum_{j=1}^{n}d(Q_{i}(j),{\hat{Q}}_{i}(j))=\frac{1}{n}\sum_{j=1}^{n}{\left(Q_{i}(j)-{\hat{Q}}_{i}(j)\right)}^{2}$. These assumptions allow us to model the encoder-decoder pair as a rate-distortion scheme of rate *R*, where the encoder is described by the mapping *f*_*n*_:**Q**_*i*_→{1,2,…,2^*nR*^}, which represents the compressed version of the vector **Q**_*i*_ of length *n* using *nR* bits, and the decoder is described by the mapping $g_{n}:\{1,2,\ldots ,2^{\text{nR}}\}\to {\hat{Q}}_{i}$, where ${\hat{Q}}_{i}=g_{n}(f_{n}(Q_{i}))$ denotes the reconstructed sequence.

With this formulation of the problem we can use some results on rate distortion theory to guide the design of QualComp. For a detailed description on rate distortion theory and proofs, please refer to [41]. We are interested in the following result:

#### Theorem 1

For an i.i.d. Gaussian vector source $X∼N(0,\Sigma_{X})$, with *Σ*_**X**_= diag$\left[\sigma_{1}^{2},\ldots ,\sigma_{n}^{2}\right]$ (i.e., independent components), the optimal allocation of *nR* bits that minimizes the MSE is given as the solution to the following optimization problem:

(1) $$\begin{array}{ll} & \underset{\rho =[\rho_{1},\cdots \;,\rho_{n}]}{\text{min}}\frac{1}{n}\overset{n}{\underset{j=1}{\sum }}\sigma_{j}^{2}2^{-2\rho_{j}}\; \end{array}$$

(2) $$\begin{array}{ll} & \text{s.t.}\overset{n}{\underset{j=1}{\sum }}\rho_{j}\leq \text{nR},\; \end{array}$$

where *ρ*_*j*_ denotes the number of bits allocated to the *j*^*th*^ component of **X**.

Next we describe how we use this result in the design of QualComp. In real data, quality scores take integer values in a finite alphabet Q, but for the purpose of modeling, we assume Q=R (the set of real numbers). Although the quality scores of different reads may be correlated, we model correlations only within a read, and consider quality scores across different reads to be independent. Thus we assume that each quality score vector **Q**_*i*_ is independent and identically distributed (i.i.d.) as *P*_**Q**_, which will be specified below.

To the best of our knowledge, there are no known statistics of the quality score vectors. However, given a vector source with a particular covariance matrix, the multivariate Gaussian is the least compressible. Furthermore, compression/coding schemes designed on the basis of Gaussian assumption, i.e., worst distribution for compression, will also be good for non-Gaussian sources, as long as the mean and the covariance matrix remain unchanged [40]. Guided by this observation, we model the quality scores as being jointly Gaussian with the same mean and covariance matrix, i.e., $P_{Q}∼N(\mu_{Q},\Sigma_{Q})$, where *μ*_**Q**_ and *Σ*_**Q**_ are empirically computed from the set of vectors ${\left\{Q_{i}\right\}}_{i=1}^{N}$. Due to the correlation of quality scores within a read, *Σ*_**Q**_ is not in general a diagonal matrix. Thus to apply Theorem 1, we need to first decorrelate the quality score vectors.

In order to decorrelate the quality score vectors, we first perform the singular value decomposition (SVD) of the matrix *Σ*_**Q**_. This allows us to express *Σ*_**Q**_ as *Σ*_**Q**_=*VSV*^*T*^, where *V* is a unitary matrix that satisfies *VV*^*T*^=*I* and *S* is a diagonal matrix whose diagonal entries *s*_*jj*_, for *j*∈[1:*n*], are known as the singular values of *Σ*_**Q**_. We then generate a new set of vectors ${\left\{{Q^{\prime }}_{i}\right\}}_{i=1}^{N}$ by performing the operation $Q_{i}^{\prime }=V^{T}(Q_{i}-\mu_{Q})$ for all *i*. This transformation, due to the Gaussian assumption on the quality score vectors, makes the components of each $Q_{i}^{\prime }$ independent and distributed as *N*(0,*s*_*jj*_), for *j*∈[1:*n*], since $Q_{i}^{\prime }∼N(0,S)$. This property allows us to use the result of Theorem 1. The number of bits alloted per quality score vector, *nR*, is a user specified parameter. Thus we can formulate the bit allocation problem for minimizing the MSE as a convex optimization problem, and solve it exactly. That is, given a budget of *nR* bits per vector, we allocate the bits by first transforming each **Q**_*i*_ into $Q_{i}^{\prime }$, for *i*∈[1:*N*], and then allocating bits to the independent components of $Q_{i}^{\prime }$. In order to minimize the MSE, we solve the following optimization problem:

(3) $$\begin{array}{ll} \underset{\rho =[\rho_{1},\cdots \;,\rho_{n}]}{\text{min}}\frac{1}{n}\overset{n}{\underset{j=1}{\sum }}s_{\text{jj}}2^{-2\rho_{j}} & \; \end{array}$$

(4) $$\begin{array}{ll} \text{s.t.}\overset{n}{\underset{j=1}{\sum }}\rho_{j}\leq \text{nR}, & \; \end{array}$$

where *ρ*_*j*_ represents the number of bits allocated to the *j*^*th*^ position of $Q_{i}^{\prime }$, for *i*∈[1:*N*], i.e., the allocation of bits is the same for all the quality score vectors and thus the optimization problem has to be solved only once. Ideally, this allocation should be done by vector quantization, i.e., by applying a vector quantizer with *Nρ*_*j*_ bits to $Q_{i}^{\prime }{\left(j\right)}_{i=1}^{N}$, for *j*∈[1:*n*]. However, due to ease of implementation and negligible performance loss, we use a scalar quantizer. Thus for all *i*∈[1:*N*], each component $Q_{i}^{\prime }(j)$, for *j*∈[1:*n*], is normalized to a unit variance Gaussian and then it is mapped to decision regions representable in *ρ*_*j*_ bits. For this we need *ρ*_*j*_ to be an integer. However, this will not be the case in general, so we randomly map each *ρ*_*j*_ to $\rho_{j}^{\prime }$, which is given by either the closest integer from below or from above, so that the average of $\rho_{j}^{\prime }$ and *ρ*_*j*_ coincide. In order to ensure the decoder gets the same value of $\rho_{j}^{\prime }$, the same pseudorandom generator is used in both functions. The decision regions that minimize the MSE for different values of *ρ* and their representative values are found offline from a Lloyd Max procedure [42] on a scalar Gaussian distribution with mean zero and variance one. For example, for *ρ*=1 we have 2^1^ decision regions, which correspond to values below zero (decision region 0) and above zero (decision region 1), with corresponding representative values −0.7978 and +0.7978. Therefore, if we were to encode the value −0.344 with one bit, we will encode it as a ‘0’, and the decoder will decode it as −0.7978. The decoder, to reconstruct the new quality scores ${\left\{{\hat{Q}}_{i}\right\}}_{i=1}^{N}$, performs the operations complementary to that done by the encoder. The decoder constructs round (*VQ*^′^+*μ*_**Q**_) and replaces the quality scores corresponding to an unknown basepair (given by the character ‘N’), by the least reliable quality value score. The steps are summarized below.

Notice that the final size is given by *nRN* plus an overhead to specify the mean and covariance of the quality scores, the length *n* and the number of sequences *N*. This can be further reduced by performing a lossless compression using a standard universal entropy code.

Since the algorithm is based on the statistics of the quality scores, better statistics would give lower distortion. With that in mind, and to capture possible correlation between the reads, we allow the user to first cluster the quality score vectors, and then perform the lossy compression in each of the clusters separately. For that we use the standard *k-means algorithm*[43], which we explain below. Notice that the total size in this case is just increased by a small amount for each of the clusters, since we do not preserve the order of the sequences. Specifically, after decoding the quality scores, we create the corresponding FASTQ file by incorporating the remaining information, i.e., the first three lines of each entry (including the header and the nucleotide sequence), and sorting the entries in alphabetical order with respect to the headers. This guarantees that related FASTQ files with paired end reads will have the same ordering after applying the lossy compression algorithm.

Finally, notice that *R*=0 is not the same as discarding the quality scores, since the decoder will not assign the same value to all the reconstructed quality scores. Instead, the reconstructed quality score vectors within a cluster will be the same, and equal to the empirical mean of the original quality score vectors within the cluster, but each quality score within the vector will in general be different.

#### Clustering

The clustering is based on the *k-means algorithm*[43], and it is performed as follows. For each of the clusters, we initialize a mean vector *V* of length *n*, with the same value at each position. The values are chosen to be equally spaced between the minimum quality score and the maximum. For example, if the quality scores go from 33 to 73 and there are 3 clusters, the mean vectors will be initialized as all 33’s, all 53’s, and all 73’s. Then, each of the quality score vectors will be assigned to the cluster that minimizes the MSE with respect to its mean vector *V*, i.e., to the cluster that minimizes $\frac{1}{n}\sum_{i=1}^{n}{\left(Q(i)-V(i)\right)}^{2}$. After assigning each quality score vector to a cluster, the mean vectors are updated by computing the empirical mean of the quality score vectors assigned to the cluster. This process is repeated until none of the quality score vectors is assigned to a different cluster, or until a maximum number of iterations is reached.

## Results and discussion

Ideally, when testing a lossy compression scheme over quality scores, one would like to know how the compression affects the results of downstream algorithms which use the quality scores. However, lossy compression of quality scores has been introduced only recently, and has not been tested extensively in that respect. In general, even without compression, there is a limited amount of work on the behavior of downstream application with respect to quality scores. Further complicating the assessment is the fact that there are no standard benchmarks, and there are very few datasets for which results (e.g., assembled genome, SNPs) were validated.

When assessing the performance of our lossy compression algorithm we consider two aspects. First is the distortion rate tradeoff achieved. That is, the MSE vs. the compression rate. This measure is independent of the downstream application and provides a framework that can be used for an evaluation of different lossy-compression algorithms. Second, we use a few publicly available datasets to test the impact of our algorithm on two downstream applications: short-read alignment and SNP detection.

### Datasets

We consider the lossy compression of three different datasets. We use two of the datasets used in [39], both generated by Solexa technology. Specifically, we consider the lossy compression of the *SRR032209* dataset [44] from a *M. musculus* species, with *N*=18828274, *n*=36 and quality scores in the range [33:67], and the *SRR089526* dataset [45] from an *H. sapiens* with *N*=23892841, *n*=48 and quality scores in the range [33:73]. We also consider a dataset from the *PhiX*[46] virus used in the control lane in the Illumina technology, where the genome from which the reads are generated is assumed to be known. This data set contains *N*=13310768 quality score sequences of length *n*=100 and values in the range [66:98]. All data sets are available for download at [47].

### Timing and memory usage

We provide estimates of the time and memory used by QualComp to compress and decompress the quality scores presented in the FASTQ files of the *PhiX, SRR03229* and *SRR089526* data introduced above. Recall that the number of reads in each file is 13310768,18828274 and 23892841, respectively, and the length of each read is 100,36 and 48, respectively. Computing the statistics needed for compression took 20, 4 and 10 minutes, respectively. However, notice that the statistics need to be computed only once. The compression time increases with the rate, varying from 0 seconds with zero rate to 9 minutes with *R*=2.5 and *PhiX* data, 3 minutes with *R*=2 and *SRR032209* data and 8 minutes with *R*=2.5 for *SRR089526* data.

The decompression time of the algorithm increases with the rate as well, and varies from 3 minutes (*R*=0) to 7 minutes (*R*=2.5) for the *PhiX* data, from 1 minute (*R*=0) to 2 minutes (*R*=2) for *SRR032209* data and from 2 minutes (*R*=0) to 4 minutes (*R*=2.5) for *SRR089526* data.

Finally, the memory usage for all the datasets during the compression and the decompression was about 16 MB, making it suitable for execution on any personal computer.

### Simulation results

#### Performance in terms of the MSE

We apply QualComp to the data sets introduced above. For each of the data sets, we run the algorithm with several rates and different number of clusters. We then compute the MSE between the uncompressed quality scores (i.e., the original quality scores) and the quality scores reconstructed by QualComp. To evaluate the performance, we consider the lossy compression schemes proposed in [39], SCALCE [35] and fastqz [34]. To that end, we run the algorithm of [39] with the options for lossy compression *LogBinning, UniBinning* and *Truncating*, for different parameters, and SCALCE with different error thresholds. We choose the option *gzip* for the final compression method in both algorithms. We also run the algorithm fastqz with different quantization levels and command “*c*”, since it offers more compression than the command “*e*” while creating the same reconstructed quality scores. For a fair comparison, we compute the number of bits per quality score employed on average by each of the algorithms and plot it versus the MSE. We do not consider the scheme proposed in [32], because we did not succeed in running the lossy compressor of quality scores presented in the SLIMGENE package. Also, the schemes proposed in [33] and Cramtools [36] employ a reference for compression, so we did not consider them.

Notice that, in our scheme, there is a small overhead due to some extra information needed for the decoder to decompress the quality scores. As a result, the actual rate (bits per quality scores) after the compression, which we denote by *R*^′^, may not be exactly equal to the parameter *R* we set when calling the program. The use of a general compression tool such as *gzip* after our compression could further decrease the size of the output file, but the reduction is very small so we do not include it in our final results. 

**Figure 1:**
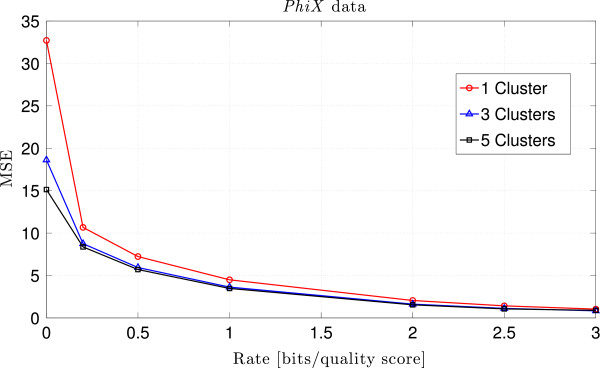
**MSE vs. compression rate for the *PhiX *dataset.** Results of QualComp when applied to the *PhiX* dataset for rates *R*={0,0.2,0.5,1,2,2.5,3}, and 1,2 and 3 clusters. As can be observed, increasing the number of clusters improves the performance of QualComp in terms of the MSE.

1. *PhiX* data:

We run our lossy compression algorithm with rates *R*={0,0.2,0.5,1,2,2.5,3}, and 1,3 and 5 clusters. Table 1 shows the MSE obtained with the different parameters, where *R* and *C* denote the rate and the number of clusters specified when running QualComp. As can be observed from these results, increasing the rate for a given number of clusters decreases the MSE, especially for small values of *R*. Similarly, increasing the number of clusters for the same rate decreases the MSE, as expected. This can be clearly seen in Figure 1. The overhead incurred by setting the number of clusters to 1,3 and 5 is 100 KB, 292 KB and 488 KB, respectively, which represents an increase in rate of 6.15×10^−4^, 1.79×10^−3^ and 3.00×10^−3^.

**Figure 2:**
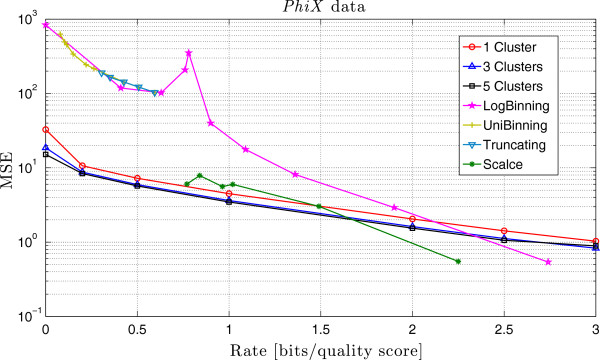
**Comparison of the MSE of different compression methods on the *PhiX *dataset.** Comparison between the MSE obtained by QualComp and the schemes proposed in [39] and SCALCE [35] for different rates. Note that for small rates QualComp presents the smallest MSE, and it achieves rates not attainable by other lossy compression algorithms.

The results obtained by running the schemes proposed in [39] and SCALCE [35] are shown in Table 2 and Table 3, respectively. No results are provided for the fastqz software [34], because it only accepts Sanger files with quality scores between 33 and 73. For ease of comparison, we plot the rate versus the MSE for all the schemes in Figure 2. As can be observed, QualComp presents a smaller MSE than that of [39] and SCALCE for the same rates in most of the cases. For rates above 2, both the *LogBinning* scheme proposed in [39] and SCALCE present a smaller MSE than QualComp with rate 3. This is due to our assumption that Q=R, instead of Q=[66:98].

As a result, QualComp may perform worse than other algorithms for rates close to those of lossless compression. However, in such a scenario (when the rate is high), the savings with lossy compression are marginal compared to a lossless compressor.

Finally, notice that in the *LogBinning* scheme proposed in [39] (as well as in SCALCE), there is no clear correlation between the number of quality scores per bin (the error threshold) and the corresponding rate and MSE. Moreover, both schemes present an MSE that is not monotonically decreasing with increasing rate. Notice also that SCALCE can not achieve rates smaller than 0.77 in this case, whereas QualComp can work at any rate. Also, the schemes *UniBinning* and *Truncating* are unable to get an MSE less than 100 with this data.

2. *M. musculus* data (*SRR032209*):

We run QualComp with rates *R*={0,0.33,0.66,1,2}, and 1,2 and 3 clusters. Table 4 shows the MSE obtained with the different parameters. *R* and *C* denote the rate and the number of clusters, and *R*^**′**^ denotes the actual rate obtained after applying the compression. The results we get are consistent with what we observed in the previous dataset. Note that the difference between *R* and *R*^**′**^ is due to the extra information (fixed) about the statistics and the number of clusters. This additional rate per read (i.e., *R*^**′**^**−***R*) becomes negligible for a large number of reads *N*, as can be observed in Table 4.

With two clusters, 24.31*%* of the quality score sequences are in one cluster and the remaining ones in the other, with the small cluster corresponding to that of quality scores with lower mean. In the case of three clusters, the division is 21.17*%*, 15.01*%* and 63.81*%*. Finally, the overheads for one, two and three clusters are 20 KB, 40 KB and 52 KB, respectively.

**Figure 3:**
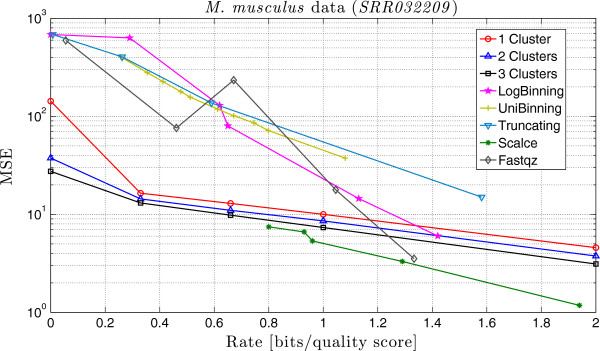
**Comparison of the MSE of different compression methods on the *M. musculus *dataset.** Comparison between the MSE obtained by QualComp and the schemes proposed in [39]**, SCALCE **[35]** and fastqz **[34]** for different rates. Note that QualComp presents the smallest MSE for small rates.**

In order to compare the performance of QualComp, we run the schemes proposed in [39]**], SCALCE [**[35] and fastqz [34], with different parameters. The corresponding rate and MSE for the different schemes are shown in Table 5 and Table 6, and in Figure 3.

As can be seen, QualComp performs well in terms of the MSE, especially for small values of *R*, as in the previous case. Notice that, as before, SCALCE can not work at small rates. Similarly, fastqz can not work below rate 0.05, and the MSE is not decreasing as a function of the rate. Among the schemes proposed in [39], *LogBinning* performs better for higher rates, and *Truncating* for smaller rates.

3. *H. sapiens* data (*SRR089526*):

For this dataset, we run QualComp with a choice of rates given by *R*={0,0.25,0.5,1,2} and 1,2 and 3 clusters. The obtained MSE for the different parameters is shown in Table 7. The overhead for the choice of 1,2 and 3 clusters is 28 KB, 56 KB and 84 KB, respectively, which represents an increment (i.e., *R*^**′**^**−***R*) of 2×10^**−4**^**, 4×10**^**−4**^** and 6×10**^**−4**^. As before, increasing the number of clusters decreases the MSE, and this difference is more noticeable for small values of *R*. For example, with *R*=0, we have a reduction of more than 77*%* in the MSE when dividing the data into three clusters instead of one, whereas the reduction for *R*=2 is around 43*%*.

**Figure 4:**
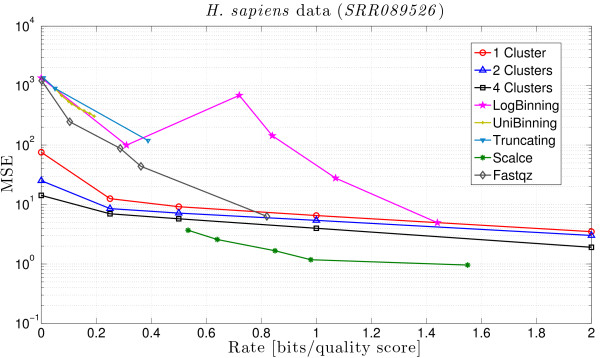
**Comparison of the MSE of different compression methods on the *H. sapiens *dataset** Comparison between the MSE obtained by QualComp and the schemes proposed in [39], SCALCE [35] and fastqz [34] for different rates.

Finally, Table 8 shows the rate and the MSE obtained when applying the schemes proposed in [39] for different parameters, and Table 9 shows the results for SCALCE and fastqz. We see from these results and Figure 4, that QualComp can work at rates not attainable by other algorithms and that it has the lowest MSE for small rates. This is again consistent with our observations from the previous datasets.

**Table 1:** **MSE results of QualComp when applied to the *PhiX *dataset**

**R**	**C**	**MSE**
0	1	32.71
0	3	18.62
0	5	15.13
0.2	1	10.67
0.2	3	8.75
0.2	5	8.37
0.5	1	7.23
0.5	3	5.94
0.5	5	5.70
1.0	1	4.49
1.0	3	3.63
1.0	5	3.47
2.0	1	2.05
2.0	3	1.62
2.0	5	1.54
2.5	1	1.42
2.5	3	1.12
2.5	5	1.06
3.0	1	1.03
3.0	3	0.89
3.0	5	0.83

MSE obtained by our lossy compression algorithm for different rates *(R)* and number of clusters *(C)* with the *PhiX* dataset. As can be observed, increasing the number of clusters decreases the MSE for the same rate.

**Table 2:** **Rate and MSE obtained by running the algorithm proposed in **[39]** on the *PhiX *dataset**

**LogBinning**	**R**	**MSE**		**UniBinning**	**R**	**MSE**		**Truncating**	**R**	**MSE**
60	0	836.54		2	0.08	629.25		33	0.30	189.63
32	0.78	352.20		4	0.10	493.59		40	0.35	165.27
30	0.76	207.50		6	0.11	452.24		60	0.42	142.76
25	0.63	102.14		10	0.15	339.58		70	0.50	122.08
20	0.41	118.67		20	0.22	243.96		80	0.59	103.19
15	0.9	39.86		30	0.26	215.86		90	0.59	103.19
10	1.09	17.67		60	0.42	142.76				
6	1.36	8.13		70	0.50	122.08				
4	1.90	2.92		80	0.59	103.19				
2	2.74	0.54		90	0.59	103.19				

MSE obtained by the *LogBinning, UniBinning* and *Truncating*** schemes proposed in **[39]** with different parameters.**

**Table 3:** **Rate and MSE obtained by running SCALCE **[35]** on the *PhiX *dataset**

**Error threshold**	**R**	**MSE**
60	0.84	7.87
70	0.77	6.06
80	0.77	6.06
90	1.02	5.99
100	0.96	5.59
40	1.49	3.03
20	2.25	0.55
0	2.95	0

MSE obtained by SCALCE with different error thresholds.

**Table 4:** **MSE results of QualComp when applied to the *M. musculus * dataset**

**R**	**C**	**R’**	**MSE**
0	1	0	143.0
0	2	0	37.58
0	3	0	27.49
0.33	1	0.3343	16.46
0.33	2	0.3420	14.39
0.33	3	0.3459	13.09
0.66	1	0.6685	12.94
0.66	2	0.6811	11.00
0.66	3	0.6825	9.82
1.00	1	1.0026	10.01
1.00	2	1.0153	8.59
1.00	3	1.0278	7.35
2.00	1	2.0051	4.58
2.00	2	2.0177	3.76
2.00	3	2.0300	3.12

MSE obtained by our lossy compression algorithm for different rates *(R)* and number of clusters *(C)* on the *M. musculus* dataset. *R’* denotes the actual rate obtained after compression. Note that the effective rate *R’*, while still close to *R*, grows with the number of clusters.

**Table 5:** **Rate and MSE obtained by running the algorithm proposed in **[39]** on the *M. musculus *dataset**

**LogBinning**	**R**	**MSE**		**UniBinning**	**R**	**MSE**		**Truncating**	**R**	**MSE**
60	0	684.29		5	0.25	405.14		33	0.01	684.29
34	0.29	632.13		10	0.35	279.63		40	0.26	404.92
26	0.65	80.160		17	0.41	226.08		50	0.58	137.08
17	0.62	129.42		26	0.47	178.60		60	1.58	15.01
10	1.13	14.51		34	0.51	157.10		70	3.24	0.00
5	1.42	6.03		60	0.61	118.58				
				70	0.67	101.53				
				80	0.74	85.92				
				90	0.74	85.92				
				100	0.79	71.82				
				200	1.08	37.53				

MSE obtained by the *LogBinning, UniBinning* and *Truncating* schemes proposed in [39] for different parameters.

**Table 6:** **Rate and MSE obtained by running SCALCE **[35]** and fastqz **[34]** on the *M. musculus * dataset**

**Scalce**			**fastqz**			
**Error threshold**	**R**	**MSE**	**Quantization**	**R**	**MSE**	
90	0.80	7.47	70	0.05	596.15	
80	0.80	7.47	50	0.05	596.15	
100	0.93	6.62	40	0.05	596.15	
60	0.96	5.36	30	0.67	234.36	
40	1.29	3.31	20	0.46	76.19	
20	1.94	1.18	10	1.04	17.83	
0	2.65	0	5	1.33	3.53	
			1	2.59	0	

MSE obtained by SCALCE with different error thresholds and by fastqz with different quantization levels.

**Table 7:** **MSE results of QualComp when applied to the *H. sapiens * dataset**

**R**	**C**	**MSE**
0	1	75.64
0	2	25.21
0	3	17.32
0.25	1	12.55
0.25	2	8.53
0.25	3	7.26
0.50	1	9.18
0.50	2	7.17
0.50	3	5.90
1.00	1	6.53
1.00	2	5.42
1.00	3	4.16
2.00	1	3.50
2.00	2	3.02
2.00	3	1.99

MSE obtained by our lossy compression algorithm for different rates *(R)* and number of clusters *(C)* with the *H. sapiens* dataset.

**Table 8:** **Rate and MSE obtained by running the schemes proposed in **[39]** on the *H. sapiens *dataset**

**LogBinning**	**R**	**MSE**	**UniBinning**	**R**	**MSE**	**Truncating**	**R**	**MSE**
60	0	1346.35	5	0.05	895.92	33	0.01	1346.35
40	0.72	684.87	10	0.07	680.27	44	0.05	895.87
30	0.31	99.59	20	0.10	538.43	60	0.39	119.04
20	0.84	143.35	30	0.11	494.93			
10	1.07	27.80	40	0.13	413.58			
5	1.44	4.95	60	0.15	375.73			
			70	0.17	339.76			
			80	0.19	305.66			
			90	0.19	305.66			

MSE obtained by the *LogBinning, UniBinning* and *Truncating* algorithms proposed in [39] for different parameters.

**Table 9:** **Rate and MSE obtained by running SCALCE **[35]** and fastqz **[34]** on the *H. sapiens * dataset**

**Scalce**			**fastqz**		
**Error threshold**	**R**	**MSE**	**Quantization**	**R**	**MSE**
100	0.5339	3.69	70	0.02	1208.02
90	0.64	2.58	50	0.02	1208.02
80	0.64	2.58	40	0.02	1208.02
60	0.85	1.67	20	0.10	246.37
40	0.98	1.18	30	0.28	87.99
20	1.55	0.96	10	0.36	43.86
0	2.04	0	5	0.82	6.31
			1	2.04	0

MSE obtained by SCALCE with different error thresholds and by fastqz with different quantization levels.

#### Impact on downstream applications

We test QualComp on two downstream applications: short-read alignment and SNP detection. For alignment, we use the *Bowtie* algorithm [10], and compare the results obtained with the original *PhiX* FASTQ file to those obtained after applying QualComp^**a**^. We chose this dataset because the virus *PhiX* has a known assembled genome [48], which we assume to be correct. This allows us to consider that a perfect mapping, i.e., a mapping with no mismatches, is correct and more valuable than a mapping that has mismatches between the read and the region where it maps in the genome. We present the alignment results in Table 10.

**Table 10:** **Alignment accuracy on the *PhiX *dataset with and without compression**

		** Mismatches**					
**bits/quality score**	**0**	**1**	**2**	**3**	**≥ 4**	**Unmapped**	**Size (MB)**
		** Original**					
2.95	11315113	1179411	237852	89385	141300	347707 (2.61%)	468.096
		** 1 Cluster**					
0	11315113	1178443	237493	67493	298	511928 (3.84%)	0.097
0.20	11315113	1179059	237691	86662	90262	401981 (3.01%)	32.097
0.50	11315113	1179153	237726	88051	100677	390048 (2.93%)	80.097
1.00	11315113	1179233	237766	88771	109950	379935 (2.85%)	159.097
2.00	11315113	1179304	237801	89177	120269	369104 (2.77%)	318.097
2.50	11315113	1179318	237813	89250	123610	365664 (2.74%)	397.097
		** 3 Clusters**					
0	11315113	1179104	237763	79908	100618	398262 (2.99%)	0.285
0.20	11315113	1179221	237793	86486	120835	371320 (2.78%)	32.411
0.50	11315113	1179268	237799	87857	124371	366360 (2.75%)	81.185
1.00	11315113	1179298	237816	88621	128182	361738 (2.71%)	159.985
2.00	11315113	1179346	237827	89108	132675	356699 (2.67%)	318.585
2.50	11315113	1179362	237835	89204	134221	355033 (2.66%)	398.385
		** 5 Clusters**					
0	11315113	1179057	237742	83060	110348	385448 (2.89%)	0.476
0.20	11315113	1179239	237796	86437	121236	370947 (2.78%)	32.551
0.50	11315113	1179283	237799	87858	124886	365829 (2.74%)	80.606
1.00	11315113	1179321	237813	88664	128682	361175 (2.71%)	160.376
2.00	11315113	1179363	237828	89146	133300	356018 (2.67%)	319.270
2.50	11315113	1179364	237833	89230	134703	354525 (2.66%)	400.176

Alignment results of *Bowtie* with the original *PhiX* FASTQ file and the ones reconstructed by QualComp, with different parameters. The first column specifies the rate, and the remaining ones the number of reads that are mapped to the reference genome with 0, 1, 2, 3 and more than 4 mismatches, and those that did not map. Last column shows the total size after compression. To compute the size of the quality scores in the original FASTQ file, we apply SCALCE [35] with lossless compression. Note that the number of reads that map with zero mismatches remain constant for all the choices of rate and number of clusters, and is equal to that of the original file.

As this table shows, increasing the rate and the number of clusters results in a mapping which is closer to that of the original FASTQ file. Also, notice that the number of reads that map with zero mismatches is constant with all the tested parameters. The main differences between the original FASTQ file and the reconstructed ones (after applying QualComp) are for alignments with high number of mismatches. Even with 0 bits per quality score, the difference in mapping is about 1.23*%*, 0.38*%* and 0.28*%* for 1, 3 and 5 clusters, respectively, whereas the savings in size are significant. With 3 clusters, for example, the savings are 467 MB. With 2 bits per quality score and 3 clusters, the difference in mapping is 0.06*%* and in size 149 MB.

In order to see how lossy compression affects the SNP detection for this dataset, *PhiX*, we run the alignment algorithm BWA [11] followed by Samtools [49] for the original and the compressed files with one, three and five clusters and different rates. However, no SNPs were detected for any of the files (including the original FASTQ file), and therefore no results are provided. Therefore, in this case, the output of the SNP detection is unaffected by the use of QualComp.

We also perform alignment with BWA [11] followed by SNP detection with Samtools [49] for the *M. musculus* data. Specifically, we use as a reference chromosome one of the Mus Musculus reference genome release, known as *MGSCv37*. We compare the output of Samtools using the original FASTQ file as an input with the reconstructed ones after applying QualComp. We show the results of SNP detection in Table 11, with one, two and three clusters and rates *R*={0,0.20,0.33,0.66,1,2}. We did not report results for higher number of clusters because we observed no noticeable improvements. We also omit the alignment results since BWA does not use the quality scores for alignment.

**Table 11:** **SNP calling on the *M. musculus * dataset with and without compression**

				**One cluster**			
**R**	**MSE**	**T.P.**	**F.P**	** F.N.**	**Selectivity (%)**	**Sensitivity (%)**	**Size (MB)**
0	143.0	11217	2033	1810	84.66	86.11	0.019
0.20	19.16	12585	1159	442	91.57	96.61	16.17
0.33	16.46	12602	1120	380	91.84	97.07	26.68
0.66	12.94	12669	998	358	92.70	97.25	53.34
1.00	10.01	12656	875	371	93.53	97.15	80.82
2.00	4.58	12733	594	294	95.54	97.74	161.62
				**Two clusters**			
**R**	**MSE**	**T.P.**	**F.P**	** F.N.**	**Selectivity (%)**	**Sensitivity (%)**	**Size (MB)**
0	37.58	12086	1534	941	88.73	92.77	0.039
0.20	16.42	12644	1184	383	91.44	97.06	16.19
0.33	14.39	12655	1107	372	91.95	97.14	26.70
0.66	11.00	12669	985	358	92.78	97.25	53.36
1.00	8.59	12687	830	340	93.85	97.39	80.84
2.00	3.76	12751	606	276	95.46	97.88	161.64
				**Three clusters**			
**R**	**MSE**	**T.P**.	**F.P**	** F.N.**	**Selectivity (%)**	**Sensitivity (%)**	**Size (MB)**
0	27.49	12048	1219	979	90.81	92.48	0.050
0.20	14.89	12638	1108	389	91.93	97.01	16.21
0.33	13.09	12645	1070	382	92.19	97.06	26.98
0.66	9.82	12646	909	381	93.29	97.07	53.91
1.00	7.35	12685	776	342	94.23	97.37	80.85
2.00	3.12	12730	554	297	95.83	97.72	161.65

We compare the SNPs detected by Samtools with the original FASTQ file and those obtained with the compressed files, using QualComp with one, two and three clusters and different rates. In all cases, reads were aligned first using the BWA algorithm. T.P., F.P. and F.N. stand for true positive (detected both with the original FASTQ file and the reconstructed one), false positive (detected only with the reconstructed FASTQ file) and false negative (detected only with the original FASTQ file), respectively. The selectivity parameter is computed as T.P./(T.P. + F.P.), and sensitivity as T.P./(T.P. + F.N.). Note that already for R = 0.2 the sensitivity is above 96% and the selectivity is above 91%.

As can be seen, both the selectivity and the sensitivity parameters (refer to Table 11 for definitions) increase with the rate, as expected. Also, since increasing the rate decreases the MSE, a lower MSE yields a performance closer to that of using the original FASTQ file. For example, with two clusters and 0.66 bits per quality score, we get a selectivity of 92.78*%* and a sensitivity of 97.25*%*. With three clusters and 1 bit per quality score, we increase both the selectivity and sensitivity to 94.23*%* and 97.37*%*, respectively.

Finally, we perform the same simulations with the *H. sapiens* data. We use as a reference chromosome one of the human reference genome release *hg19*, also known as *GRCh37*. We show the results of SNP detection in Table 12, with one, two and three clusters, and rates *R* = {0,0.2,0.25,0.5,1,2}. As in the previous case, lower MSE (and therefore higher rate) yields a performance closer to that of the original FASTQ file. For example, with two clusters and 0.25 bits per quality score, we get 92.25*%* selectivity and 97.64*%* sensitivity, and with three clusters and 0.5 bits per quality score, we get 93.06*%* selectivity and 97.82*%* sensitivity. Also, increasing the number of clusters yields a better selectivity. This, together with the previous results, supports the use of the MSE as a metric for evaluating any lossy compression algorithm.

**Table 12:** **SNP calling on the *H. sapiens *dataset with and without compression**

				**One cluster**			
**R**	**MSE**	**T.P.**	**F.P**	** F.N.**	**Selectivity (%)**	**Sensitivity (%)**	**Size (MB)**
0	75.64	54945	11560	5482	82.62	90.93	0.027
0.20	13.95	58806	5952	1621	90.81	97.32	27.37
0.25	12.55	58881	5707	1546	91.16	97.44	34.20
0.50	9.18	59078	5022	1349	92.17	97.77	68.38
1.00	6.53	59349	4541	1078	92.89	98.22	136.74
2.00	3.50	59628	3814	799	93.99	98.68	273.45
				**Two clusters**			
**R**	**MSE**	**T.P.**	**F.P**	** F.N.**	**Selectivity (%)**	**Sensitivity (%)**	**Size (MB)**
0	25.21	51007	5010	9420	91.05	84.41	0.054
0.20	9.09	58955	4949	1472	92.25	97.56	27.39
0.25	8.53	59002	4951	1425	92.25	97.64	34.23
0.50	7.17	59188	4784	1239	92.52	97.94	68.41
1.00	5.42	59400	4559	1027	92.87	98.30	136.76
2.00	3.02	59601	3718	826	94.12	98.63	273.48
				**Three clusters**			
**R**	**MSE**	**T.P.**	**F.P.**	** F.N.**	**Selectivity (%)**	**Sensitivity (%)**	**Size (MB)**
0	17.32	52922	4686	7505	91.87	87.58	0.082
0.20	7.80	58913	4823	1514	92.43	97.49	27.42
0.25	7.26	58977	4766	1450	92.52	97.60	34.26
0.50	5.90	59111	4411	1316	93.06	97.82	68.44
1.00	4.16	59247	4041	1180	93.61	98.05	136.79
2.00	1.99	59589	3262	838	94.81	98.61	273.51

We compare the SNPs detected by Samtools with the original FASTQ file and those obtained with the compressed files, using QualComp with one, two and three clusters and different rates. For more details see Table 11.

### Setting the compression rate **R**

The performance of the algorithm in terms of the MSE and its effect on downstream applications is highly data dependent, and therefore each user should decide which rate to use based on their storage capabilities and the accuracy required for the reconstructed quality scores.

However, based on the results we obtained with the three datasets we experimented with, we can suggest some general guidelines on how to select *R* effectively. The trade-off between rate and performance for QualComp is best for small values of *R*, i.e., for the same increment in rate, the improvement in performance is higher for small rates (see Additional file 1: Figure S1). For example, although *R*=0 gives the most savings in terms of storage, with *R*=0.05 and two clusters, the sensitivity of SNP calling on the *M. musculus* dataset jumps from 92.77*%* to 96.42*%*, and from 84.41*%* to 96.30*%* for the *H. sapiens* data (see Additional file 1: Table S1 and Table S2). Furthermore, increasing the rate to 0.5 increases the sensitivity for the *M. musculus* data only slightly to 97.16*%*, but increases the storage space drastically (by around 900 percent) from 4.07 MB (*R*=0.05) to 40.44 MB (*R*=0.5). Similar observations hold for the *H. sapiens* data.

Looking at the MSE, we observe a similar behavior. Specifically, the slope of the MSE with respect to the rate is more pronounced for small values of *R*. For example, for the *PhiX* dataset (see Additional file 1: Table S3), we observe the biggest relative decrease from *R*=0 (MSE =18.62) to *R*=0.05(MSE =11.73), which is a 37 percent improvement. Then, for *R*=0.3 we get an MSE of 7.58, and for *R*=0.5 an MSE of 5.94. Similarly, for the *H. sapiens* dataset we observe a factor of two reduction in the MSE, from 25.21 with *R*=0 to 13.55 with *R*=0.05. For *R*=0.2 we get an MSE of 9.09 and further increasing the rate to 0.5 gives an MSE of 7.17.

We therefore propose to set *R*=0.2 as a starting point, since it offers better performance than *R*=0 while still offering major savings in size (see Additional file 1: Table S4). For example, while *Gzip* can compress the dataset *PhiX* to 592 MB, QualComp produces a file of size 32.09 MB for rate *R*=0.2 and one cluster (94% reduction). Such savings are very significant for large-scale metagenomics projects, which generate massive datasets. Similarly, *Gzip* reduces the *H. sapiens* dataset to 389 MB, while QualComp can compress the file to 27.42 MB (*R*=0.2 and 3 clusters), which represents a reduction in size of 93%.

As for the number of clusters, the results show a notable improvement when switching from one to three clusters. For example, with *R*=0.2 the alignment results of *Bowtie* on the *PhiX* dataset show 3.01*%*,2.78*%* and 2.78*%* unmapped reads with one, three and five clusters, respectively. Similar results are obtained for different choices of *R*. For the *H. sapiens* data and *R*=0.2, we get an MSE of 13.95 with one cluster, 9.09 with two clusters and 7.80 with three clusters. Therefore we suggest setting the number of clusters to 3.

QualComp does take longer to run compared to other programs (see Additional file 1: Table S4), however most of it is spent on the computation of the statistics and the clustering, which do not depend on the rate. For example, computing the statistics for the *H. sapiens* (*SRR089526*) and the *M. musculus* (*SRR032209*) datasets took approximately 10 and 4 minutes (both with 1 and 3 clusters), respectively. Clustering the *H. sapiens* and the *M. musculus* datasets to three clusters took about 24 and 20 minutes, respectively. However, notice that the clustering only affects the compression and not the decompression time, which is approximately a few minutes for the considered datasets (and therefore for FASTQ files with similar sizes). In general, the decompression time scales linearly with the number of reads.

Moreover, note that when compressing a dataset with different rates, the clustering process and the computation of the statistics need to be performed only once. Furthermore, for large sets of sequencing data, one needs to perform clustering and estimation of the statistics (mean and covariance) only on a small subset (can be thought of as the training set) of the data. One can then use those estimates to compress every subsequent read. This can be done by first assigning the read to a cluster and then using the proposed compression scheme. This will lead to a significant reduction in the compression time.

In summary, the proposed scheme can offer major savings in storage space while still enabling accurate reconstruction of the quality scores. We suggest the use of small rates (between 0.05 and 0.5) and 3 clusters. We stress here that our study of downstream applications assumes read sequences from a single organism. However, the scale of savings from our scheme becomes more significant for larger datasets such as those used for metagenomics studies [3,4]. For the scale of data reported in [3] (268 gigabases) our methods can reduce the storage requirements for the quality values to 1.67 GB using a rate of 0.05 or 16.75 GB (*R*=0.5) (as opposed to around 83 GB achieved by a lossless compressor algorithm if we assume a rate of 2.5 bits per quality score). Similarly for the data sets in [4] (576.7 gigabases), we could do with 3.6 GB of storage for rate of 0.05 and 36 GB for rate 0.5, instead of 180 GB for lossless compression.

## Conclusions

To tackle the problem of storage and dissemination of genomic data, we have developed QualComp, a new algorithm for the lossy compression of the quality scores presented in a FASTQ file. One advantage of the proposed method with respect to other lossy compression algorithms is that it allows the user to specify the rate (number of bits per quality score) prior to compression. This choice should be made according to the storage availability of each user and the amount of accuracy required for the reconstructed quality scores. Given a model for the quality scores and using theoretical results on rate-distortion, QualComp optimally allocates the bits in order to minimize the MSE. We then compare several lossy compression schemes by looking at the MSE versus the rate, thus making the comparison independent of downstream applications that use quality scores. We show that our algorithm results in better MSE, compared to alternative schemes, for small rates. QualComp can also work at rates not attainable with other algorithms, and presents an MSE that decreases monotonically as a function of the rate.

We also test QualComp on two downstream applications (short-read alignment and SNP calling), showing that little is compromised in performance, while the file size is reduced significantly after compression. However, to better understand how the lossy compression algorithm affects the downstream applications, more simulations with several datasets and full sequence analysis workflow should be performed, once such benchmarks become available.

## Availability

**Software name:** QualComp

**Software home page:**https://sourceforge.net/projects/qualcomp/

**Programming languages:** C

**License:** web server freely available without registration

Restrictions to use by non-academics: on request

## Endnote

^**a**^**We also tried the alignment programs BWA [**[11] and MAQ [9]. However, BWA does not use the quality scores for the alignment and we were unable to run MAQ in our system.

## Abbreviations

MSE: Mean squared error; NGS: Next generation sequencing; SRA: Sequence read archive; SNP: Single nucleotide polymorphism; SVD: Singular value decomposition; TP: True positives; FP: False positives; FN: False negatives; KB: KiloByte.

## Competing interests

The authors declare that they have no competing interests.

## Authors’ contributions

HA, MC, DB and TW conceived of the project idea. IO and MC implemented the algorithm in C, and performed the simulations. GY designed the performance evaluation protocols. All authors contributed to the design and analysis and interpretation of the results. IO and MC wrote the first version of the manuscript, and IO, HA, MC, TW and GY were involved in revising it. All authors read and approved the final manuscript.

## Supplementary Material

Additional file 1 **Choosing the rate R.** We present additional simulations of QualComp on the three datasets to illustrate some of the trade-offs in choosing *R* (pdf extension). Click here for file
