# Supplementary material for: QualComp: a new lossy compressor for quality scores based on rate distortion theory
Source: BMC Bioinformatics. 2013 Jun 8;14:187. doi: 10.1186/1471-2105-14-187 (PMC3698011; doi:10.1186/1471-2105-14-187)
Supplement: Additional file 1 — Choosing the rate R. We present additional simulations of QualComp on the three datasets to illustrate some of the trade-offs in choosing R (pdf extension). [file 1471-2105-14-187-S1.pdf]

Additional File 1 - Choosing the rate R

To illustrate some of the trade-offs in choosing R, we present additional simulations in this document. The general trend we would like to highlight is that while higher rates usually lead to better performance (in terms of MSE, selectivity and sensitivity), most of the gains are already achieved with small rates (e.g. $R = 0.20$), where the savings in disk space are most substantial.

Table 1 - SNP calling on the M. musculus dataset (SRR032209) with and without compression

A detailed analysis of SNP calling, as in Table 11, over a refined range of rate values.

Two clusters							
R	MSE	T.P.	F.P	F.N.	Selectivity (%)	Sensitivity (%)	Size (MB)
0	37.58	12086	1534	941	88.73	92.77	0.039
0.05	22.73	12561	1345	466	90.33	96.42	4.07
0.10	19.27	12596	1285	431	90.74	96.69	8.11
0.15	17.60	12620	1212	407	91.24	96.88	12.15
0.20	16.42	12644	1184	383	91.44	97.06	16.19
0.25	15.53	12648	1143	379	91.71	97.09	20.23
0.30	14.80	12668	1128	359	91.82	97.24	24.27
0.33	14.39	12655	1107	372	91.95	97.14	26.70
0.35	14.14	12663	1093	364	92.05	97.21	28.31
0.40	13.55	12646	1071	381	92.19	97.08	32.35
0.45	13.00	12634	1043	393	92.37	96.98	36.40
0.50	12.47	12657	1043	370	92.39	97.16	40.44
0.66	11.00	12669	985	358	92.78	97.25	53.36
1.00	8.59	12687	830	340	93.85	97.39	80.84
2.00	3.76	12751	606	276	95.46	97.88	161.64
Three clusters							
R	MSE	T.P.	F.P	F.N.	Selectivity (%)	Sensitivity (%)	Size (MB)
0	27.49	12048	1219	979	90.81	92.48	0.050
0.05	19.37	12535	1217	492	91.15	96.22	4.09
0.10	17.10	12599	1160	428	91.56	96.71	8.13
0.15	15.83	12609	1134	418	91.74	96.79	12.17
0.20	14.89	12638	1108	389	91.93	97.01	16.21
0.25	14.16	12635	1092	392	92.04	96.99	20.25
0.30	13.50	12645	1087	382	92.08	97.06	24.30
0.33	13.09	12645	1070	382	92.19	97.06	26.98
0.35	12.90	12632	1054	395	92.50	96.96	28.33
0.40	12.35	12632	1027	395	92.48	96.96	32.37
0.45	11.82	12642	997	385	92.69	97.04	36.41
0.50	11.33	12628	983	399	92.77	96.93	40.45
0.66	9.82	12646	909	381	93.29	97.07	53.91
1.00	7.35	12685	776	342	94.23	97.37	80.85
2.00	3.12	12730	554	297	95.83	97.72	161.65

Table 2 - SNP calling on the H. sapiens dataset (SRR089526) with and without compression

A detailed analysis of SNP calling, as in Table 12, over a refined range of rate values.

Two clusters							
R	MSE	T.P.	F.P	F.N.	Selectivity (%)	Sensitivity (%)	Size (MB)
0	25.21	51007	5010	9420	91.05	84.41	0.054
0.05	13.55	58195	5292	2232	91.66	96.30	6.88
0.10	11.45	58674	5321	1753	91.68	97.09	13.72
0.15	10.09	58865	5050	1562	92.09	97.41	20.56
0.20	9.09	58955	4949	1472	92.25	97.56	27.39
0.25	8.53	59002	4951	1425	92.25	97.64	34.23
0.30	8.13	59048	4942	1379	92.27	97.71	41.06
0.35	7.84	59090	4894	1337	92.35	97.78	47.90
0.40	7.60	59134	4874	1293	92.38	97.86	54.74
0.45	7.38	59129	4869	1298	92.39	97.85	61.57
0.50	7.17	59188	4784	1239	92.52	97.94	68.41
1.00	5.42	59400	4559	1027	92.87	98.30	136.76
2.00	3.02	59601	3718	826	94.12	98.63	273.48
Three clusters							
R	MSE	T.P.	F.P	F.N.	Selectivity (%)	Sensitivity (%)	Size (MB)
0	17.32	52922	4686	7505	91.87	87.58	0.082
0.05	11.05	58451	4977	1976	92.15	96.73	6.91
0.10	9.57	58784	5111	1643	92.00	97.28	13.75
0.15	8.54	58839	4950	1588	92.24	97.37	20.58
0.20	7.80	58913	4823	1514	92.43	97.49	27.42
0.25	7.26	58977	4766	1450	92.52	97.60	34.26
0.30	6.89	58996	4661	1431	92.68	97.63	41.09
0.35	6.60	59031	4563	1396	92.82	97.69	47.93
0.40	6.35	59016	4497	1411	92.92	97.66	54.76
0.45	6.12	59065	4468	1362	92.97	97.75	61.60
0.50	5.90	59111	4411	1316	93.06	97.82	68.44
1.00	4.16	59247	4041	1180	93.61	98.05	136.79
2.00	1.99	59589	3262	838	94.81	98.61	273.51

Table 3 - MSE and file sizes of QualComp when applied to the PhiX dataset

MSE and file sizes obtained by our lossy compression algorithm for different rates (R) and three clusters (C) with the *PhiX* dataset.

R	C	MSE	Size (MB)
0	3	18.62	0.28
0.05	3	11.73	8.21
0.10	3	10.36	16.15
0.15	3	9.45	24.08
0.20	3	8.75	32.41
0.25	3	8.13	39.95
0.30	3	7.58	47.88
0.35	3	7.10	55.82
0.40	3	6.67	63.75
0.45	3	6.29	71.68
0.50	3	5.94	81.18
1.00	3	3.63	159.98
2.00	3	1.62	318.58
2.50	3	1.12	398.38
3.00	3	0.89	476.31

Table 4 - Comparison of file sizes and running times with different compression methods

We apply SCALCE, Gzip and our lossy compression algorithm on the *PhiX*, *H. sapiens* (*SRR089526*) and *M. musculus* (*SRR032209*) datasets. For QualComp we report the results both without clustering ($C = 1$) and with clustering ($C = 3$). In both cases the rate R is set to 0.20.

	PhiX	SRR089526	SRR032209
Original file	1.3 GB	1.1 GB	665 MB
Lossless Compression			
Gzip	592.00 MB	389.00 MB	277.00 MB
Time Gzip	2 min	1.5 min	1 min
Scalce	468.09 MB	278.90 MB	214.12 MB
Time Scalce	8.5 min	5 min	2 min
Lossy Compression (QualComp)			
$C = 1$ and $R = 0.20$	32.09 MB	27.37 MB	16.17 MB
Compression	3.5 min	2 min	1 min
Statistics	22 min	9.5 min	4 min
$C = 3$ and $R = 0.20$	32.41 MB	27.42 MB	16.21 MB
Compression	4 min	2 min	1 min
Statistics	25 min	10 min	4 min
Clustering	60 min	24 min	20 min

Figure 1 - Selectivity and sensitivity of SNP calling as a function of the rate over the H. sapiens dataset (SRR089526)

We assess the selectivity and sensitivity of SNP calling with Samtools on the *H. sapiens* dataset, compressed using QualComp with different rates and 2 clusters (a) and 3 clusters (b). Definitions of selectivity and sensitivity are as in Table 11. Red dashed lines represent the slope at different points of the

curve. Note that the most significant improvement is obtained in the range $0 - 0.2$. Hence, we suggest setting the default value of R to 0.2.

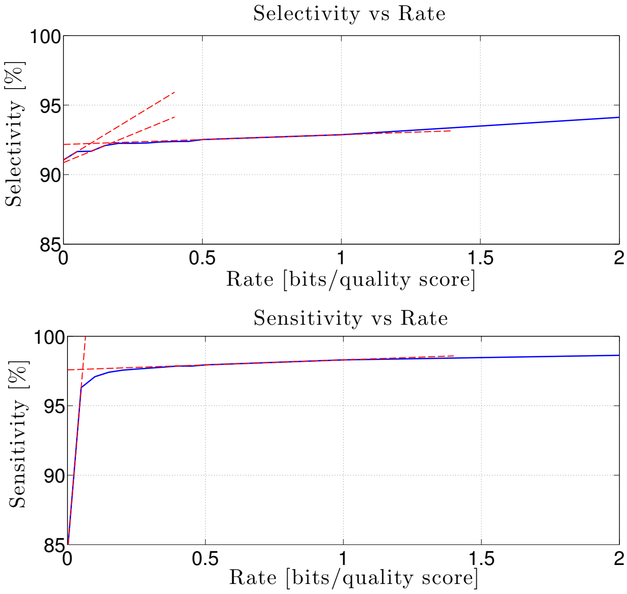

(a) QualComp applied with two clusters

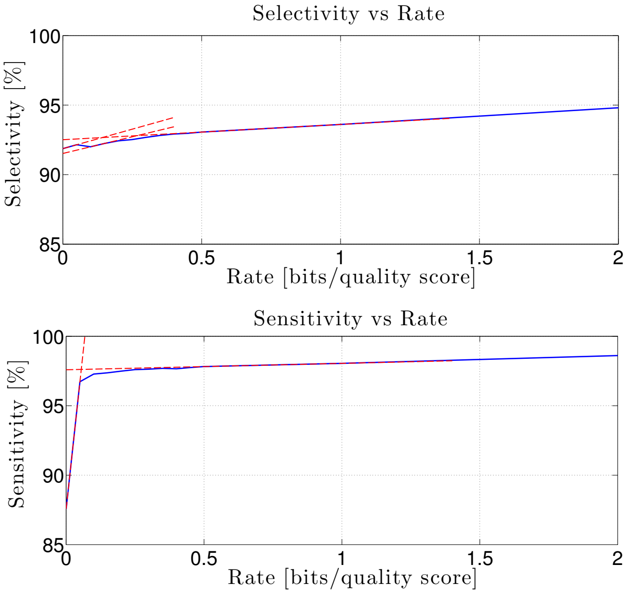

(b) QualComp applied with three clusters
